# A Case of Triple Respiratory Coinfection: COVID-19, Enterovirus, and Parainfluenza

**DOI:** 10.7759/cureus.72949

**Published:** 2024-11-03

**Authors:** Muhammad H Bangash, Shehar B Awais, Chaudhary A Cheema, Andrew J Luker, Steven Prado, Abdul Waheed

**Affiliations:** 1 Department of Family Medicine, WellSpan Good Samaritan Hospital, Family Medicine Residency Program, Lebanon, USA; 2 Department of Family Medicine, Creighton University, School of Medicine, Phoenix, USA; 3 Department of Internal Medicine, Creighton University, School of Medicine, Phoenix, USA; 4 Department of Family and Community Medicine, Creighton University, School of Medicine, Phoenix, USA; 5 Department of Family Medicine, Dignity Health Medical Group, Gilbert, USA

**Keywords:** coinfection, covid-19, enterovirus, parainfluenza, superinfection

## Abstract

We report a case of a 34-year-old female who presented to the emergency department with fever, nausea, vomiting, and diarrhea following a suspected foodborne illness. She tested positive for COVID-19, human parainfluenza virus type 4, and enterovirus/rhinovirus in the hospital. She subsequently developed hypoxia, hypotension, and sepsis. Blood work revealed leukocytosis and elevated inflammatory markers. Imaging of her abdomen showed fluid-filled bowel loops suggesting acute gastroenteritis and colitis of infectious or inflammatory etiology. She was initially treated with antibiotics for a suspected bacterial infection. However, her cultures resulted negative and her symptoms slowly improved with supportive care including probiotics and a low-fat diet. Due to her persistent shortness of breath related to her COVID-19 infection, she was started on a course of oral prednisone due to the anti-inflammatory effects of steroids. She was discharged home in stable condition with close outpatient follow-up. This study highlights the clinical challenges of managing multiple viral infections, particularly with concurrent COVID-19.

## Introduction

Respiratory viral infections represent one of the most common clinical problems in all age groups globally [1]. The complexity of patient care increases significantly when infected with multiple viruses, occasionally leading to synergistic effects. This synergism ultimately leads to a more severe manifestation of the disease [2]. Coinfections are usually defined as concurrent infections whereas superinfections are those that follow the primary infection. Notable examples of coinfection include the co-occurrence of influenza A and SARS-CoV-2 causing severe, prolonged pneumonia. Additionally, human parainfluenza virus (HPIV) type 2 has also been shown to proliferate the growth of influenza [3,4]. However, triple respiratory coinfections are rare, with only 0.001% of patients testing positive for COVID-19, influenza, and respiratory syncytial virus (RSV) at the same time [5].

The emergence of COVID-19, caused by the SARS-CoV-2 virus, has significantly impacted global health, presenting a spectrum of symptoms that include fever, cough, nausea, vomiting, and anosmia. In severe cases, infections can escalate to acute respiratory distress syndrome (ARDS) and other complications, particularly in older adults and individuals with pre-existing comorbidities [3]. The symptoms of HPIV in adults are dependent on the following types: types 1 and 2 are known to cause the common cold and croup, and types 3 and 4 tend to cause more severe infections like bronchiolitis and pneumonia [6]. Additionally, the enterovirus genus can be split into rhinovirus species and non-rhinovirus species. While rhinoviruses are common causes of upper respiratory infections, both groups can cause respiratory symptoms like cough and congestion. All these three types of viruses can be spread through airborne droplets. Despite the significant burden posed by these viral pathogens, there is currently no vaccine available for viruses like HPIV or enteroviruses (except polio, which has been eradicated in the United States) [7]. Given the overlapping symptoms and disease course, understanding the relationship between multiple viral infections that coinfect individuals is critical. The case reported here offers insight into the diagnosis, management, and outcomes of patients with multiple viral coinfections by examining one such instance of triple respiratory coinfection with COVID-19, HPIV, and enterovirus.

## Case presentation

A 34-year-old female with a medical history significant for attention deficit hyperactivity disorder (ADHD), well-controlled major depressive disorder (MDD), and generalized anxiety disorder (GAD) presented from home to the emergency department (ED) with acute nausea, vomiting, and diarrhea. She was admitted for acute gastroenteritis. She reported eating fruit salad from Walmart the night before and developing symptoms shortly after that. She reported 8-10 episodes of black tarry watery stools before progressing to intractable frequency with incontinence. She reported that she was experiencing palpitations, dizziness, chills, and fever while being in the ED. She denied sick contacts, travel, or recent use of antibiotics. She was, however, seen at an urgent care four days prior with fever and cough, where she had tested positive for COVID-19, SARS-CoV-2, parainfluenza type IV, and enterovirus/rhinovirus (Table 1). Her chest x-ray at the time was unremarkable.

**Table 1:** Results of the viral panel.

Adenovirus	Not detected
Chlamydia pneumoniae	Not detected
COVID-19 SARS-CoV-2	Detected abnormal
Coronavirus	Not detected
Influenza A	Not detected
Influenza B	Not detected
Metapneumovirus	Not detected
Mycoplasma pneumoniae	Not detected
Parainfluenza	Detected abnormal
Parainfluenza, type 1	Not detected
Parainfluenza, type 2	Not detected
Parainfluenza, type 3	Not detected
Parainfluenza, type 4	Detected abnormal
Enterovirus/rhinovirus	Detected abnormal
Respiratory syncytial virus	Not detected
Bordetella pertussis	Not detected
Bordetella parapertussis	Not detected

Lab work revealed leukocytosis with a white blood cell (WBC) count of 12.5 K/µL with neutrophilic dominance, elevated C-reactive protein (CRP) and procalcitonin levels, normal erythrocyte sedimentation rate (ESR), urea and creatinine levels, and lactate levels. The stool was negative for *Clostridioides difficile *toxin by polymerase chain reaction (PCR) and Giardia antigen. Table 2 summarizes the results of this bloodwork while Table 3 summarizes the results of the stool studies.

**Table 2:** Blood work results at the time of admission to the hospital. CRP: C-reactive protein; INR: normalization ratio; eGFR: estimated glomerular filtration rate; ALT: alanine transferase; AST: aspartate transferase; BUN: blood urea nitrogen; CO_2_: carbon dioxide; SGPT: serum glutamate pyruvate transaminase; Protime: prothrombin time

Lab parameter	Normal range	Lab result
WBC	4.0-11.0 K/µL	12.5 K/µL
RBC	3.80-5.34 M/µL	5.05 M/µL
Hemoglobin	11.5-15.5 g/dL	14.7 g/dL
Hematocrit	34.5-47.0%	45.6%
Platelets	140-400 K/µL	327 K/µL
Neutrophils absolute	1.70-7.80 K/µL	11.54 K/µL
Lymphocytes absolute	1.00-4.80 K/µL	0.38 K/µL
Monocytes absolute	0.00-1.00 K/µL	0.4 K/µL
Eosinophils absolute	0.00-0.45 K/µL	0.09 K/µL
Basophils absolute	0.00-0.20 K/µL	0.03 K/µL
Glucose	70-99 mg/dL	91 mg/dL
Sodium	135-145 mmol/L	136 mmol/L
Potassium	3.5-5.3 mmol/L	3.9 mmol/L
Chloride	98-107 mmol/L	105 mmol/L
CO_2_	21-31 mmol/L	23 mmol/L
Anion gap	3-11 mmol/L	8 mmol/L
BUN	7-25 mg/dL	15 mg/dL
Creatinine	0.60-1.20 mg/dL	0.71 mg/dL
Calcium	8.6-10.3 mg/dL	8.9 mg/dL
Total protein	6.4-8.9 mg/dL	7.5 mg/dL
Albumin	3.5-5.7 g/dL	4.1 g/dL
Alkaline phosphatase	34-104 IU/L	113 IU/L
AST	13-39 IU/L	19 IU/L
ALT (SGPT)	7-52 IU/L	15 IU/L
Total bilirubin	0.3-1.0 mg/dL	0.9 mg/dL
eGFR	≥60.0 mL/min/1.73 m^2^	>90.0 mL/min/1.73 m^2^
Lactate	0.5-2.0 mmol/L	0.5 mmol/L
Sedimentation rate	0-20 mm/h	16 mm/h
CRP	≤10.0 mg/L	50.9 mg/L
Procalcitonin	<0.25 ng/mL	0.46 ng/mL
Protime	9.4-12.4 s	11.4 s
INR	0.8-1.2	1.1
Lipase	11-82 IU/L	49 IU/L
Blood culture	-	No growth at 120 h

**Table 3:** Results of the stool studies. Ag: antigen; EIA: enzyme immunoassay; WBC: white blood cell

Lab parameter	Normal range	Lab result
Fecal leukocytes	No WBCs present	No WBCs present
Giardia antigen	Not detected	Not detected
Cryptosporidium antigen	Not detected	Not detected
*C. diff *PCR	Not detected	Not detected
Occult blood, stool	Negative	Positive abnormal
Ova and parasite	Negative	No ova and parasites seen
Salmonella and Shigella culture	Not detected	Not detected
Campylobacter species AG EIA	Not detected	Not detected
Shiga toxins, EIA	Not detected	Not detected
Calprotectin	<50 µg/g normal, 50-120 µg/g borderline, >120 µg/g elevated	69 µg/g

**Figure 1:**
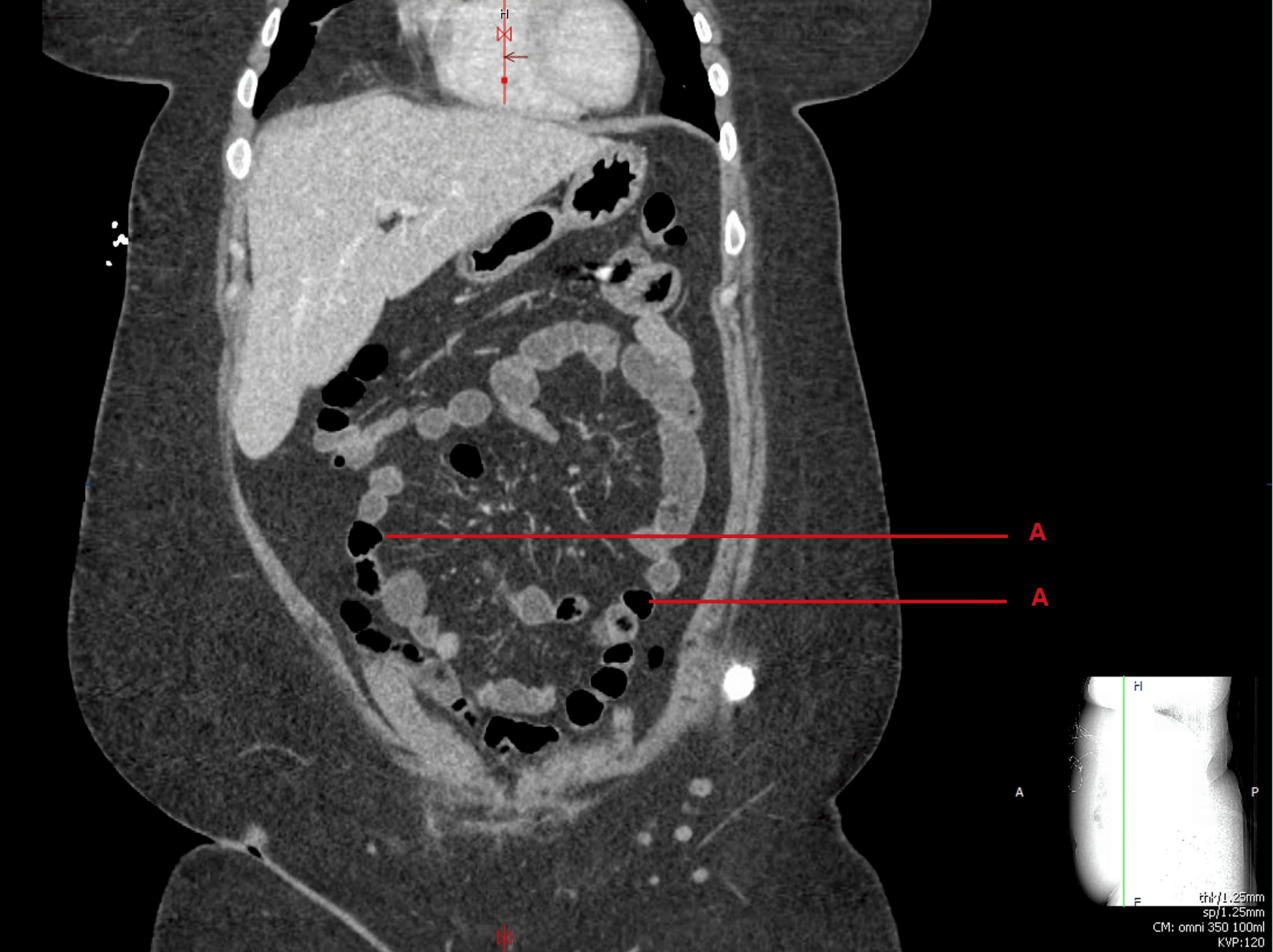
CT abdomen/pelvis on day 1 of admission showing bowel loops with air-fluid levels and colonic fluid accumulation (indicated by the red line marking A).

**Figure 2:**
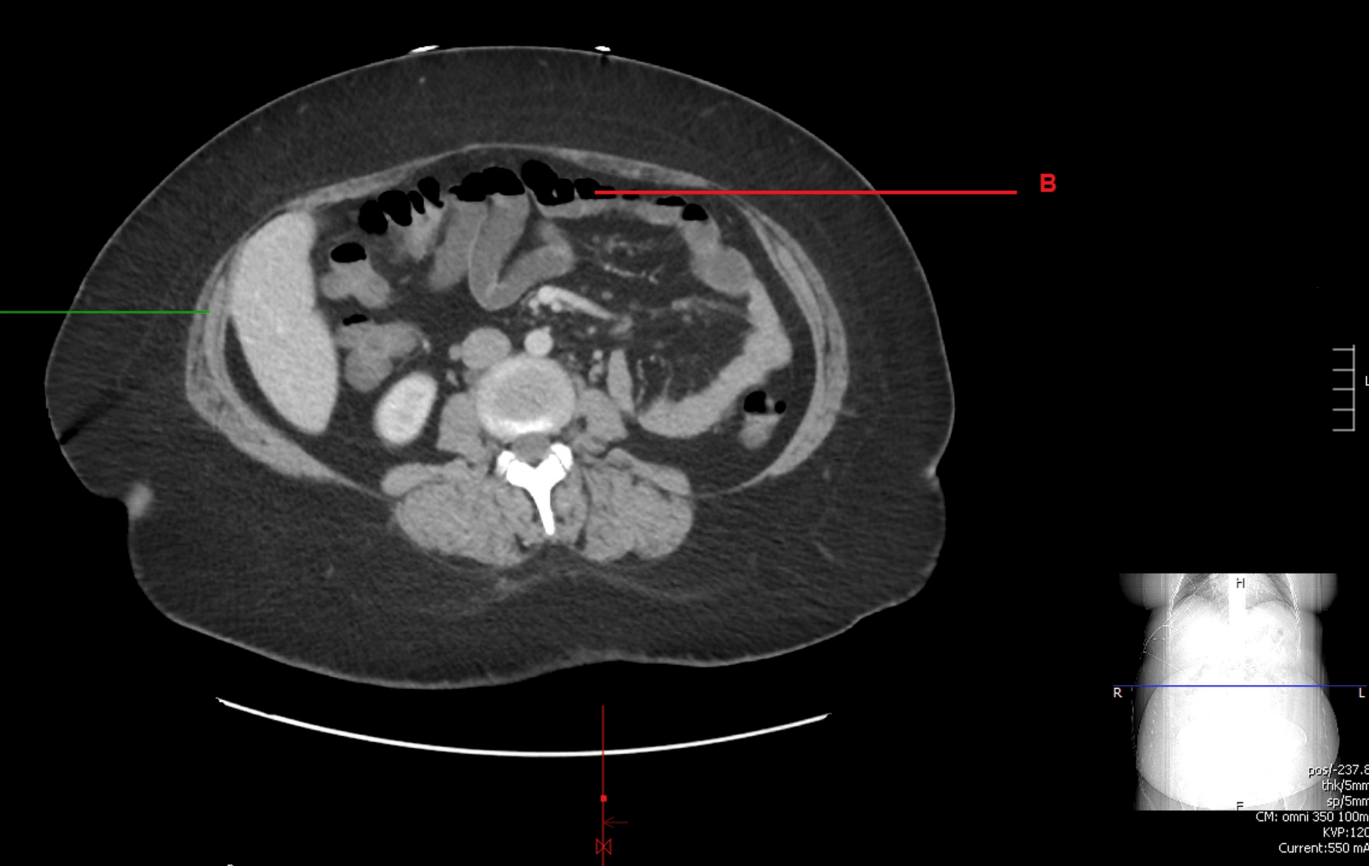
CT abdomen/pelvis with contrast showing fluid-filled bowel loops and colonic fluid (indicated by the red line marking B).

CT abdomen revealed small bowel and colonic fluid suggesting acute gastroenteritis and colitis of infectious or inflammatory etiology (Figures 1, 2). Repeat chest chest x-ray was normal as well.

In the ED, she was hypotensive with a mean arterial pressure (MAP) of 53; therefore, she received fluid resuscitation until her blood pressure normalized. She met sepsis criteria based on leukocytosis, and tachycardia with the likely source being a gastrointestinal pathogen. She was started on intravenous levofloxacin 750 mg daily, intravenous metronidazole 500 mg three times daily for suspected colitis on the day of admission along with intravenous ondansetron for nausea. Leukocytosis resolved the next day; however, the patient continued to have watery diarrhea with no improvement for three days. Stool and blood cultures were negative. Antibiotics were discontinued in view of continued diarrhea on day three and the patient was started on probiotics and a low-fat diet. Her symptoms improved the next day, and she began to have semi-formed stools; however, she reported some continued difficulty breathing. She was given oral prednisone to help with breathing and inflammatory response. On day four of admission, her initial symptoms had resolved completely and she was discharged home on an oral prednisone course of five days.

## Discussion

This case highlights the possible clinical implications of multiple viral infections occurring concurrently in a patient. Previous meta-analyses indicate that there is an increase in both severity of symptoms and odds of mortality in COVID-19 patients who had another concurrent viral infection. Patients with concurrent viral infections were more likely to develop symptoms of dyspnea compared to those infected with COVID-19 alone. It was found that among 16,643 COVID-19 patients screened within 48 hours of diagnosis, approximately 5% tested positive for another virus [8]. Another meta-analysis by Lansbury et al. estimated that approximately 3% of hospitalized COVID-19 patients were also infected with another respiratory virus, with influenza and RSV being the most common [9].

These findings also emphasize the importance of screening COVID-19 patients for additional respiratory viral infections. Identifying coinfections early can help clinicians better manage symptoms, such as dyspnea, and potentially improve patient outcomes. There are very few, if any, documented cases of adults experiencing more than two simultaneous viral infections [10,11]. Most studies have reported more than two viral coinfections in children with bronchitis, and studies indicate that an increased number of coinfections correlates with longer hospital stays [12]. A study from Poland, though somewhat dated, highlights the occurrence of multiple viral infections in immunocompromised patients or those undergoing immunosuppressive therapy [13]. However, our patient was healthy with no significant comorbidities, making this case uncommon and not previously reported in the literature. Treatment plans for COVID-19 patients with coinfections can become complicated. For example, while steroid treatments are generally contraindicated in influenza patients due to their association with increased mortality and hospital-acquired infection rates, these treatments have been beneficial for COVID-19 patients due to their anti-inflammatory properties [14,15]. Therefore, there is a need to establish a uniform management plan for patients who are simultaneously infected with both COVID-19 and another virus. This may be challenging since reported cases of COVID-19 patients with two concurrent viral infections are limited.

Finally, this case highlights the need to inform patients of preventative measures to reduce their chances of becoming infected with respiratory viruses, particularly during winter months. Practicing proper hand hygiene, which includes frequent handwashing and hand disinfection, has been shown to limit the person-to-person transmission of COVID-19 [16]. Additionally, patients should be educated on the benefit of receiving yearly vaccination for COVID-19 and influenza, which has been shown to further decrease both rates of transmission and severity of both diseases [17].

## Conclusions

In this case, we discussed the rare occurrence of triple viral coinfection in a young patient, which led to both respiratory and gastrointestinal symptoms. Given the limited reports on such cases, further research is needed to better understand the nature of COVID-19 coinfections involving two or more viruses. Emphasizing preventive measures and establishing guidelines for managing multiple concurrent infections with COVID-19 is essential for reducing rates of transmission and improving patient outcomes.
